# Climate services for society: origins, institutional arrangements, and design elements for an evaluation framework

**DOI:** 10.1002/wcc.290

**Published:** 2014-05-28

**Authors:** Catherine Vaughan, Suraje Dessai

**Affiliations:** 1 Sustainability Research Institute and Centre for Climate Change Economics and Policy, School of Earth and Environment, University of LeedsLeeds, UK; 2 International Research Institute for Climate and Society, the Earth Institute, Columbia UniversityPalisades, NY, USA

## Abstract

Climate services involve the generation, provision, and contextualization of information and knowledge derived from climate research for decision making at all levels of society. These services are mainly targeted at informing adaptation to climate variability and change, widely recognized as an important challenge for sustainable development. This paper reviews the development of climate services, beginning with a historical overview, a short summary of improvements in climate information, and a description of the recent surge of interest in climate service development including, for example, the Global Framework for Climate Services, implemented by the World Meteorological Organization in October 2012. It also reviews institutional arrangements of selected emerging climate services across local, national, regional, and international scales. By synthesizing existing literature, the paper proposes four design elements of a climate services evaluation framework. These design elements include: problem identification and the decision-making context; the characteristics, tailoring, and dissemination of the climate information; the governance and structure of the service, including the process by which it is developed; and the socioeconomic value of the service. The design elements are intended to serve as a guide to organize future work regarding the evaluation of when and whether climate services are more or less successful. The paper concludes by identifying future research questions regarding the institutional arrangements that support climate services and nascent efforts to evaluate them.

## INTRODUCTION

Humans have always faced climate-related risk. Throughout history, departures from the seasonal rhythms of climate have meant the difference between wealth and poverty, health and sickness, and even life and death.^1^ As we look to the future, human welfare will increasingly be tied to the extent to which we are able to manage the risks and opportunities associated with a changing climate.^2^,^3^ Climate services, which provide timely, tailored information and knowledge to decision makers (generally in the form of tools, products, websites, or bulletins), are seen as an important part of improving our capacity to manage climate-related risk.

Although climate services are often used in conjunction with information about the weather, climate services are also distinct from weather services, which describe the state of the atmosphere at a given place and time.^4^ Rather, climate services convey information about average weather, using the analysis of time series data to estimate trends, departures from average conditions, and low-probability events on timescales from seasons to centuries.^5^,^6^ Climate services are also distinct from climate research and observations: the former focuses on serving user needs while the latter aims to further our understanding of the climate system.

The aim of climate services is to provide people and organizations with timely, tailored climate-related knowledge and information that they can use to reduce climate-related losses and enhance benefits, including the protection of lives, livelihoods, and property (for more regarding definitions, see Box 1).

**BOX 1 TERMINOLOGY**

While the diversity of actors engaged in climate service provision increases the coverage and hopefully the quality of climate services, it also challenges our ability to talk about and distinguish between different kinds of ‘services’.

In current parlance, for instance, the German Climate Service Center—a free-standing organization that engages in a range of different activities—is described as a ‘climate service’ in just the same way that the provision of forecast information to the Red Cross through an online mapping tool is. Similarly, Ethiopia's Climate and Health Working Group—which meets to discuss issues related to climate impacts on health—is described as a ‘service’ in the same way that climate information bulletins or decision-support tools are.

Given this situation, the range of actors, entities, and activities that fall under this term can sometimes be confusing. For the purposes of this paper, we will use the following terms:

**Climate service coordinating bodies**, including the Global Framework for Climate Services, work to increase connections between climate information users and providers and to support the development of climate services in particular contexts.
**Climate service users** employ climate information and knowledge for decision making; they may or may not participate in developing the service itself. In some cases, climate information users may also pass information along to others, making them both users and providers.
**Climate service providers** supply climate information and knowledge. Climate service providers may operate on international, national, regional, or local levels and in a range of different sectors; they may be public or private, or some mixture of both.
**Climate impact monitoring groups** meet to monitor and discuss evolving climate impacts and implications of forecasts for decision making in particular contexts, especially with regard to health (e.g., Climate and Health Working Groups that monitor the incidence of climate-sensitive diseases) and food security. They generally include decision makers, sectoral experts, and representatives from practitioner communities.
**Climate services** involve the direct provision of knowledge and information to specific decision makers. They generally involve tools, products, websites, or bulletins.

Touted as an important part of the adaptation agenda, climate services have received a great deal of attention in recent years. Humanitarian organizations, government offices, international agencies, and the private sector have focused attention on climate services as a way to improve climate risk management and increase resilience, focusing in particular on the use of historical information,^7^ seasonal forecasts,^8^–^11^ and long-term climate projections.^12^–^16^ As a result, climate services currently exist at local, national, regional, and international scales and in a range of different sectors, including agriculture, health, forestry, fisheries, transport, tourism, disaster risk reduction, water resources management, and energy.^17^,^18^

But while climate services hold the promise of improving economic and social well-being, there has been relatively little evaluation of their performance.^19^–^22^ As a result, it is difficult to assess the extent to which individual climate services and/or climate services in general live up to this promise. This leaves climate service users, providers, and funding agencies with very little information about the quality and relative value of climate services. In this context, organizations find it difficult to make informed decisions about where to devote time and resources to improve the development, delivery, and use of climate knowledge and information for societal benefit.

Improving our understanding of the role and relative contribution of climate services is thus a critical step in enhancing our ability to manage climate-related risk. To what extent should climate services be supported? In what contexts are they successful? How should we define success? What can be done to improve performance? While the answers to these questions are still opaque, the design elements of an evaluation framework will begin to improve our understanding of the utility of climate services, and the factors that make them more or less successful; it will also help us to parse the range of evaluation methodologies that may be used by different actors interested in different aspects of the performance of climate services. This information is also critical for future research on evaluation that will ultimately improve decisions about where and when to invest in these new endeavors.

As an initial contribution to this discussion, our paper reviews the academic literature to describe the development of climate services, paying particular attention to the range of institutional arrangements that support them and the historical context in which they have emerged; it also proposes design elements for an evaluation framework through which we can begin to assess the relative contribution of climate services to the benefit of society.

The article begins with a section on the *Historical Context* in which climate services have developed. The next section on *Current Services* and *Delivery Structures* explores the various institutional arrangements that have developed to provide climate services across scales. The following section illustrates some of the challenges associated with *Identifying Benefits of Climate Services*: *the Case of Agriculture*. The next section synthesizes literature on the use of climate information in order to propose *Design Elements for an Evaluation Framework of Climate Services*. The final section provides *General Conclusions and Areas for Future Research*.

## HISTORICAL CONTEXT

While the term ‘climate services’ has come into favor fairly recently, the research and observational programs that helped to build our current scientific capacity to offer user-oriented services date back more than a century. This section reviews some of the critical developments that formed the cornerstone for this scientific capacity, and notes that many of these programs were formed with the idea of providing benefit to society.

The World Meteorological Organization—together with its predecessor, the International Meteorological Organization (IMO)—has worked to create a framework for international cooperation on climate research and data exchange for societal benefit since late 19th century. More recently, the WMO convened a series of international climate conferences that have been instrumental both in advancing research and observational capacity, and in generating and sustaining interest in climate-services activities.

The first of these was the first World Climate Conference (WCC 1), held in 1979 as a ‘world conference of experts on climate and mankind’. Citing the ‘all-pervading influence of climate on human society and on many fields of human endeavour’, the conference statement called on the nations of the world to take advantage of man's present knowledge of climate, and to take steps to improve that knowledge. It also encouraged efforts to predict and prevent potential man-made changes in climate that might be adverse to the well-being of humanity.^23^

To further these goals, WCC 1 called for the creation of a World Climate Programme (WCP) to improve our understanding of the climate system and its impact on society.^24^,^25^ In doing so, the WCP both led and responded to activities around the world. The U.S. National Climate Program, for instance, was created in 1978 to ‘assist the Nation and the world to understand and respond to natural and man-induced climate processes and their implications’.^26^–^28^ Immediately following the creation of the WCP, the Australian Bureau of Meteorology began exploring opportunities to create a National Climate Centre to serve as the national focus for all matters relating to Australian climate and climate data.^29^ In 1988, the UK government announced its intention to create the Met Office Hadley Centre to focus climate science research.^30^

These and many other organizations fostered the activities of the WCP throughout the 1980s. Within the WCP, the creation of the Tropical Ocean-Global Atmosphere research program led, in the late 1980s, to the development of predictive models of the El Niño Southern Oscillation and a relative breakthrough in our understanding of the climate system.^31^,^32^ Shortly thereafter, the Intergovernmental Panel on Climate Change (IPCC) was created to assess the impacts of increasing greenhouse gas concentrations on the climate.^33^ The second World Climate Conference (WCC 2) was convened in 1990 to review both the first 10 years of the WCP and the IPCC's first assessment report.

WCC 2 led to the creation, in 1992, of the United Nations Framework Convention on Climate Change and the Global Climate Observing System. Importantly, it also endorsed a ‘Climate Agenda’ which focused the attention of governments on improving climate observation, prediction, impact assessment, and services.^34^ While formally endorsed, the Climate Agenda did not gather steam until developments within the United Nations Framework Convention on Climate Change—and the second (1995) and third (2001) IPCC assessment reports—led people to the conclusion that addressing climate change would require a mix of mitigation and adaptation strategies.^35^

These developments focused attention on the need to provide comprehensive scientific information to support such actions; it also underscored the continuing importance of earlier initiatives to support the development of climate information and service delivery.^35^ The WMO organized two conferences that addressed these issues—‘Living with Climate Variability and Change’, in 2006,^36^ and ‘Secure and Sustainable Living: Social and Economic Benefits of Weather, Climate, and Water Services’, in 2007^37^—before its 15th Congress called for a third World Conference (WCC 3). Held in 2009, WCC 3 endorsed the concept of a Global Framework for Climate Services (GFCS) to strengthen production, availability, delivery, and application of science-based climate prediction and services, particularly in developing countries.^38^

It is important to note the radical improvements in climate science that took place between WCC 1 and WCC 3. Over these 30 years, new technologies—including satellites, radar, telecommunications, and supercomputing—helped scientists to dramatically increase their understanding of the climate system.^39^–^41^ As a result, increasingly skilled predictions of climate phenomena, such as the El Niño Southern Oscillation, contributed to the production of seasonal-to-interannual climate forecasts that are significantly better than climatology.^32^,^39^ Long-term climate projections also improved, as General Circulation Models—models that describe the main interactions between various components of the climate system—have been continually refined and extended, allowing for better representation of the effect of greenhouse gases on the atmosphere, an improved description of the Earth surface and atmospheric properties.^42^

The ability to produce better information about future climates naturally led to questions about how to use that information. The use of climate forecasts was a marked improvement over previous efforts at climate-informed decision making, which generally took into account long-term means of relevant climate variables.^43^ At the same time, society became increasingly aware of its vulnerability to climate-related impacts. In the face of global change, government planning departments, development agencies, investment banks, and private companies have begun to seek out information that can help protect themselves and their constituents.^44^,^45^ An increase in the cost and frequency of climate-related disasters has also prompted disaster relief organizations and national-level decision makers to demand information they can use to help reduce disaster-related risk.^46^,^47^

In this context, climate scientists have engaged with a range of users to produce and tailor information to specific decision-making contexts. Scientists around the world are now working to produce climate information on timescales from seasons to decades and to contextualize this information for sectors as diverse as agriculture, health, transportation, water management, and disaster risk reduction; representatives of these sectors have also attempted to draw climate knowledge and information into their operations.^43^,^48^ To date, climate services focus primarily on forecasting forthcoming seasons to inform decision making; projecting long-term trends to guide policy making and strategic planning; and monitoring and predicting climate-related hazards for disaster risk management.^43^

Unfortunately, the process of developing climate services has not been easy.^10^,^21^, ^49^–^51^ In many cases, the connections between climate information users and providers are weak or nonexistent.^52^ Even in cases in which these connections do exist, climate information providers often do not fully understand the contexts in which decisions are being made.^53^ As a result, information is provided in a format that prospective users find difficult to understand and/or incorporate into decision making.^54^–^56^ While the impact of this may be neutral across socioeconomic groups in some situations, in other cases the inappropriate use of (or inability to use) climate information can increase users' risk exposure and lead to bad decisions.^57^

These challenges have shifted the focus of both scientists and decision makers to holistic solutions derived from cross-disciplinary and participatory user-oriented research.^58^ In this way, climate scientists and service providers now strive to work closely with sectoral experts, practitioners, and policy makers in a process of joint problem solving. In theory at least, the ‘co-production’ of climate services leads to services that are more effective, more usable, and more suited to users needs.^57^ ^59^–^61^

The Global Framework for Climate Services, implemented in 2012 after a period of consultation,^62^ engages with this context. In this sense, the GFCS is both a product of 30 years of effort on the part of the WMO and many others, and a direct response to on-going developments in science and society. The GFCS attempts to create a structure to support better, more informed decisions—with the ultimate goal of saving lives, protecting the environment, and improving economic development. To do this, the GFCS seeks to create a framework to coordinate and promote activities that support the development of climate services around the world.

The GFCS engages these issues through information systems; observations and monitoring; research, modeling, and prediction; capacity building; and the creation of user interface platform.^62^,^63^ The GFCS has focused initial efforts on developing countries and four priority areas (agriculture and food security, disaster risk reduction, health, and water resources), though it is expected to expand to other countries and sectors over time. While the GFCS currently draws funds from the WMO and several voluntary contributions, additional investment will be required if the GFCS is to build infrastructure and capacity and address existing funding gaps.^64^

## CURRENT SERVICES AND DELIVERY STRUCTURES

While the GFCS has garnered a great deal of attention of late, it is just one of a range of activities which engage the public and private sectors at global, national, regional, and local scales. In the context of climate services, climate service providers work with users to contextualize scientific knowledge, enabling climate information to be created and tailored to specific decision contexts.^66^ In this way, climate services operate at the boundary between climate science, policy, and practice.^67^,^68^ Across scales, the institutions that support climate services seek to create a structure in which credible, salient, and legitimate information can be explored and defined by climate scientists and decision makers alike.^69^,^70^ Though an assessment of the relative merit of various institutional arrangements is beyond the scope of this paper, a brief overview of efforts at these various scales is provided in this section; more information is also available in Medri et al^71^.

### International Service Structures

In addition to the WMO, a number of organizations with global reach have begun to incorporate climate services into their repertoire. Together with the International Federation of Red Cross and Red Crescent Societies, the Netherlands Red Cross established the Red Cross Climate Centre in 2002 in order to better connect the scientific and humanitarian communities and improve the application of scientific knowledge about climate change to the early warning of disasters, health programs, and awareness raising.^72^ While the Climate Centre is based in the Netherlands, it provides climate services to Red Cross Red Crescent Societies around the world. An example of a Red Cross climate service is found in Box 2. International agencies including the Food and Agricultural Organisation, World Food Programme, Oxfam, and the World Health Organisation have also engaged in the production and delivery of relevant climate information to constituents.^73^–^77^

**BOX 2. CLIMATE INFORMATION IN CONTEXT: A CLIMATE SERVICE FOR DISASTER RISK MANAGEMENT**

Climate services are targeted at a range of sectors including health, agriculture, water resources management, and disaster risk reduction; an example of a climate service developed for the humanitarian community is included below. For more examples of climate services, please consult Nicklin et al. 2012^18^ and the case studies collected by the Climate Services Partnership.^17^

An increase in the cost and severity of climate-related disasters, as well as a growing awareness of the extent to which disasters can thwart development gains, has prompted the disaster community to take a particular interest in climate services. Together with the International Research Institute for Climate and Society (IRI), the International Federation of Red Cross and Red Crescent Societies (IFRC) developed the Forecast in Context Map Room to aid disaster-related decision making.^65^

The tool presents 6-day and 3-month forecasts against local averages to help humanitarian actors understand how current climate events compare to general conditions. It also provides information on past climate, recent climate trends, and vulnerability indicators (including population density and infant mortality) on a global scale. Information is provided in nontechnical language and accompanied by recommendations for the kinds of early action activities that disaster risk managers might take based on given forecasts (e.g., ‘consider who in your region will be most affected’, ‘review your contingency plans and update as necessary’, etc.).

The tool is supported by a Help Desk feature through which disaster risk managers can consult climate scientists to discuss the meaning and implications of information that is provided. It is used by decision makers at the Red Cross and other humanitarian organizations throughout the world (Figure 1).^46^

**Figure:**
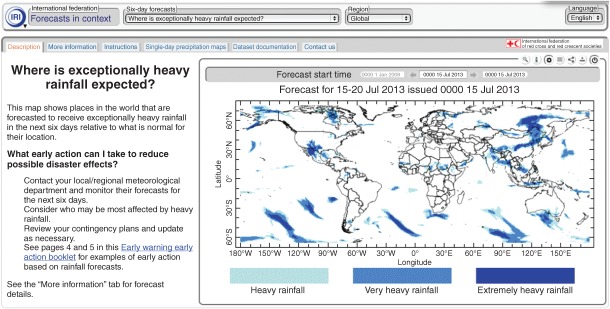
A screen shot of the IRI-IFRC Forecast in Context Tool illustrates where exceptionally heavy rainfall is expected.

The International Research Institute for Climate and Society, originally founded by the US National Oceanic and Atmospheric Administration (NOAA) to provide actionable climate information and capacity building for decision making in developing countries, also operates on international scales.^78^ In conjunction with a number of other organizations, it currently serves as the secretariat for the Climate Services Partnership, an informal international alliance which works to connect climate information users, providers, researchers, and donors around the world, and to serve as a platform in which the diverse organizations can share experiences regarding the production, delivery, and use of climate services.^17^

### National Climate Service Providers

At national scales, many state meteorological agencies provide climate services, particularly to the water and agriculture sectors.^79^,^80^ National meteorological departments also collect and manage climatological data and many produce seasonal climate outlooks.^81^ In recent years, a number of countries have consolidated these capacities into national climate service centers.

According to Miles et al.,^82^ national climate service centers meet national needs for climate information, providing an overarching and coordinated approach to managing climate observation systems and producing and disseminating information on climate and its impacts to stakeholders at federal and local levels. Centrally funded organizations in Australia,^83^ Austria,^84^ China,^85^ Finland,^86^ Germany,^87^,^88^ Italy,^89^ the Netherlands,^90^ and the UK^91^ currently strive to meet these needs, producing timely, actionable, decision-relevant information on climate variability and change and the associated environmental, economic, and societal impacts of these phenomena; the United States' Climate Prediction Center performs similar tasks.^92^ Some authors have suggested that similar organizations are needed to support development in Africa.^93^,^94^

While National Climate Centres are increasingly seen as useful,^95^,^96^ institutional arrangements that support existing organizations vary widely. While some exist as part of their national meteorological agencies, others are more independent. Some employ top-down approaches to climate information provision, while others start with bottom-up methods, including vulnerability and risk assessments. These organizations also employ different business models. While there appears to be agreement that climate services must receive some public funding in order to ensure the intellectual, economic, and political independence of groups generating knowledge, many climate service centers rely to some extent on the private sector for support, though commercial opportunities to date have been limited.^5^ In some developed countries, national climate service providers work to improve the provision of climate services in developing countries as well.

### Regional Climate Services

Regional climate service activities currently exist at both subnational and supranational scales. A prime example of the subnational climate services comes from the United States, where a system of Regional Climate Centers (RCCs) dates to the creation of the National Climate Program Act of 1978.^97^ Conceived as flexible and innovative institutions, RCCs respond to the fact that needs and uses for climate information occur in specific locations and settings.^98^,^99^ By operating within these specific locations, climate information providers improve connections with potential users and their understanding of local impacts.^27^,^79^

Because RCCs are located in different physiographic, economic, and climatic regions, their functions vary according to regional needs. In general, however, these organizations concentrate on acquiring and managing relevant data for the region and conducting applied climate studies, including the monitoring of anomalous conditions, the fostering and promoting of regional research, the creation of specialized products and decision-support tools. While oversight and funding is provided by NOAA's National Climatic Data Center, each RCC is also supported by the academic institution from which it operates, and from each state that agrees to be a participating member.^100^ Several RCCs enhance their funding by grants and contracts for services, data, and research projects from government and private-sector sources.^97^

Subnational climate services exist in other counties as well. The Northern German Climate Office, established in 2006 at the Helmholtz Centre's Institute for Coastal Research, provides climate information for the general public.^66^ As with RCCs in the US, the Northern Germany Climate Office produces technical information about the regional climate and localizes this knowledge in the social and cultural setting where people live.^101^ In somewhat similar fashion, the Pacific Climate Impacts Consortium, a federally registered not-for-profit organization, has produced practical information on the physical impacts of climate variability and change in the Pacific and Yukon Region of Canada since 2005.^102^

Regional climate services have also crossed state lines. In some cases, this involves regional organizations such as the African Centre for Meteorological Applications for Development (ACMAD), the AGHYRMET Regional Centre, the Caribbean Institute for Meteorology and Hydrology (CIMH), the International Centre for Research on the El Niño Phenomena (CIIFEN), the IGAD (Intergovernmental Authority on Development) Climate Prediction and Applications Centre (ICPAC), and the Latin American Observatory of Extraordinary Events (OLE2). A few of these regional climate providers have published on their experience.^103^–^105^

In other cases, groups of scientists from diverse organizations collaborate to provide regional climate assessments, including for instance, the BALTEX assessment of climate change for the Baltic Sea^106^ and the North Sea climate change assessment (NOSCCA).^107^ Since the late 1990s, Regional Climate Outlook Forums have also brought international, national, and regional experts together to produce consensus seasonal forecasts for particular areas with similar climatic conditions. As part of this process, sectoral scientists, extension agencies, and policy makers assess the implications of the outlooks on society and communication regarding appropriate actions.^108^,^110^,^111^

### Research Institutes

Academic and research organizations play a critical role in climate services, focusing primarily on data compilation, analysis, and product development, and engaging either on their own or with public- or private-sector partners. While the universities engaged in climate research and service development are too many to mention, a few examples are included here:

- The University of Cape Town's Climate Systems and Analysis Group (CSAG) has developed a Climate Information Portal which provides a wide range of users with climate information.^111^
- Researchers at the University of Southern Queensland and James Cook University worked to develop seasonal forecasts useful to Australia's Queensland Sugar Limited, the third-largest sugar supplier in the world^112^,^113^
- The Climate Impacts Group (CIG) of the University of Washington provides tools and planning advice that take into account the impacts of natural climate variability and global climate change^114^

The European Union has also funded several large-scale climate services research programs, which focus specifically on creating and providing climate information to specific European user groups.^115^–^118^ These projects engage a range of university and research organizations; in some cases, these and other climate service activities also engage with NGOs as a means to better connect with user groups.

### Private Sector Services

In increasing numbers, private-sector actors have begun providing value-added climate services.^119^,^120^ To inform long-term planning, private companies in the energy and insurance fields have created their own climate risk management teams and/or hired consultants to help them prepare for climate-related risks. A major energy player, the EDF group is now modeling climate change impacts on long-term energy demand and supply in different regions around the world.^121^,^122^ Swiss firm e-dric.ch produces forecasts of river discharge that are used for flood management and the development of sanitation systems.^123^

A number of private companies, such as Climate Risk Analysis,^124^ Predictia,^125^ Climpact,^126^ Climate Corporation,^127^ and Prescient Weather,^128^ have also sprung up to develop tools that help business and public-sector actors to more effectively manage climate-related risk. In many ways, these companies are similar to private weather service providers—using government-collected data to develop new tools and products that they sell to individual users and, in certain cases, collecting their own proprietary data as well.

### Climate Services across Scales

The number and range of actors involved in climate service production and development has increased the visibility and potential benefit of climate services. The diverse field also allows for a range of interesting partnerships between different organizations, researchers, and practitioners with rich and varied expertise. At the same time, the field is marked by relative fragmentation, which has created tensions and challenged effective service development by ensuring that data and know-how are spread over a wide range of different actors; potential users are also confused by the diverse array of institutions and products available.

This is compounded by a relative dearth of analysis of the sorts of institutional relationships that contribute to more successful climate services.^21^ While it seems intuitive that some services are more effectively provided by organizations operating on national versus regional scales, or by the public versus the private sector, our understanding of the kinds of services that are best provided by the array of actors mentioned above remains undeveloped. A detailed analysis of the trade-offs associated with international, national, and regional climate service providers is sorely needed, as is a close inspection of the appropriate roles of the public and private sector in meeting society's varying needs for climate services.

Understanding the value and relative contribution of climate services is a critical step in improving our ability to adapt to climate variability and change. By improving capacity to recognize and articulate which initiatives are successful, why, and to what extent, the evaluation of climate services can help inform adaptation decisions and guide future investments. Unfortunately, this evaluation is complicated by the fact that the benefits of such services can take many forms—and by the multiple, interacting attributes that contribute to creating these benefits when they do occur. In the following section, we consider challenges to identifying benefits of climate services in the context of agriculture.

## IDENTIFYING BENEFITS OF CLIMATE SERVICES: THE CASE OF AGRICULTURE

Benefits from climate services may take many forms and may accrue to the individual, the collective, or the environment though, in some cases, benefits that accrue at one level may cause associated losses at another.^1^,^129^ As a means of illustration, it may be useful to consider an example. With respect to agriculture, for instance, climate information can raise awareness of potential climate outcomes and/or help farmers mitigate the impact of unfavorable outcomes by allowing them the opportunity to make decisions about specific crops, the timing of planting, and the application of fertilizer. Farmers can also use advanced information to reduce the costs of preparing for extreme events that are less likely to occur.

At the same time, climate services can help farmers take advantage of climate-generated opportunities. In the case of both good and bad years, climate services can help farmers to prepare not just for extreme events, but also for less dramatic—but more common—variations. In the long term, climate information can raise average incomes above baseline levels as decision making consistently improves. Farmers may also experience nonmonetary benefits, including reduced planning time, reduced workload, or improved nutrition.

While the monetary and nonmonetary benefits may have positive impacts on the farmers’ communities at large, it also stands to reason that some actors may stand to lose (grain importers, for instance, may find markets less receptive in places in which production has increased as a result of climate services). It is important to note as well that situations in which forecasts do not turn out as expected may lead to negative outcomes for farmers and, potentially, their communities.

Climate services may also result in nonmonetary benefits to the environment. In the case of agriculture, for instance, better decision making could reduce fertilizer use, improve the efficiency of water allocation, reduce soil erosion and, ultimately, lower greenhouse gas emissions.^130^ Climate services can contribute to land management decisions outside of the agricultural sector as well, thus improving the management of protected spaces and water resources. Of course, the reverse is also true: information about the climate will likely lead actors to take decisions in their best interest, and there is no guarantee that the best interest of the individual or the organization will be the best interest of the environment or society at large.^131^

Climate services may also have value to the economy as a whole.^132^ For instance, an expansion of the use of climate information for agriculture may lead to beneficial adaptation systems across the sector, making agriculture in general more effective.^9^ This is true in other sectors as well, as climate services provide tools that help people and organizations adapt to and mitigate climate impacts that could potentially result in significant economic, societal, and environmental damages. Quantifying the monetary and nonmonetary benefits of climate services is an important step and one that is not well understood; more detailed study of these interacting benefits would help reveal when climate services are more or less desirable and the trade-offs they bring.

## DESIGN ELEMENTS FOR AN EVALUATION FRAMEWORK OF CLIMATE SERVICES

Even in cases in which the benefits associated with climate services are understood, there has been very little evaluation of their performance (a notable exception is found in McNie^133^). In this context, it is difficult to assess the extent to which individual climate services, climate service providers, and/or climate services in general live up to their promise. Improving our understanding of the role and relative contribution of climate services is thus a critical step in prioritizing investments and enhancing our ability to manage climate-related risk. A review of the literature describing the use of seasonal forecasts and long-term scenarios identifies various factors that influence the benefits and relative success of climate services.

In broad terms, these can be described as follows:

- Problem identification and the decision-making context
- Characteristics, tailoring, and communication of the climate information
- Governance, process, and structure of the climate service
- Socioeconomic value of the climate service

### Problem Identification and the Decision-Making Context

Climate services are developed to improve decision making in specific contexts, and naturally involve certain assumptions about those contexts. An agricultural climate service may assume, for instance, that climate variability is a constraint on production, and that low production is a constraint on farmers' livelihoods. To address this, the service supplies information at appropriate times, assuming that farmers who make better decisions—employing conservative strategies in good years, and investing when the likelihood of favorable conditions are high—will increase production and with it their ability to earn a profit. In many cases, this premise may be a valid one; in other cases, however, other factors (access to markets, trade agreements, etc.) may mean that the increase in production facilitated by the use of the climate service does not lead to an improvement in the farmers' livelihood.

Indeed, in many cases there has been an implicit assumption in many circles that as the technical constraints—including the characteristics and communication of the climate forecast—are removed, forecasts will allow various end users to improve planning and better manage the risks associated with climate variability.^134^,^135^ The truth is that in many contexts, the strongest impediments to forecast adoption are contextual or institutional.^136^ Conversely, certain situations make climate services more impactful than others, including the variability of the climate; the exposure to climate variability; capacity to incorporate climate information into decisions; and the cultural and individual context.^57^,^137^

In this regard, it is important to remember that climate services are not neutral. Climate information can be used to help specific users, potentially at the expense of others. Several case studies suggest that some users have greater access to forecasts than others, and that politics, ethnicity, and gender influence this.^138^ This is particularly true in cases in which asymmetric information and one-sided uncertainty about resources privilege certain members of society.^139^ Access, comprehension, and adoption rates are all important determinants of the distributional impacts of climate services.^140^ Identifying methods to assess the extent to which climate services address tractable problems, and do so in a way in which benefits targeted users, is something that has not yet been well addressed in the literature. The fact that positive and negative impacts may occur at different scales naturally complicates efforts to identify benefits of the service, as described above.

### Characteristics, Tailoring, and Communication of the Climate Information

The success of a climate service naturally depends on the quality of the climate information that underpins it. But while advances in climate science have allowed climate information providers to extend the limits of predictability beyond the traditional limits of weather forecasting, climate predictions are still far from perfect. Limitations in climate models and uncertainties in the observations that are used to drive them—along with intrinsic unpredictability in the climate system—mean that climate predictions are inherently probabilistic.^141^

The extent to which efforts to use this sort of information have been successful depends in great part on the extent to which the information that underpins it, matches users needs in terms of skill, scale, and lead-time.^142^ Efforts to assess the skill of forecasts are found throughout the literature,^143^–^145^ as are efforts to improve the temporal and spatial scale of forecasts and projections.^9^,^39^,^48^,^146^ Scientists have also worked hard to improve the lead-time of seasonal forecasts^147^ though the extent to which a mismatch between lead-time and the decision-making context is assessed is much less documented.^148^

The characteristics of the climate information involved are critically important, but not sufficient, to make climate services effective. Indeed, the technical and probabilistic nature of climate information makes it very difficult for nonexperts to interpret.^149^ As a result, climate information is most effective when tailored to meet recipients' needs in terms of response strategies, cultural traits, and specific situations.^143^ If the information is not appropriately tailored to specific decision contexts, it will not be useful to or usable by decision makers. As a result, it will not be used.

In that regard, assessing the extent to which information is appropriately tailored is important to understanding the efficacy of climate services. Three important aspects of this tailoring process are: the relevance and perceived relevance of the information; the accessibility of the information^144^,^145^; and the distributional impact of various groups, including those who may be more or less well-off.^139^

In the face of climate change, Bettencourt^150^ has also suggested that national planners need to consider what is likely to happen in the future, and the uncertainties surrounding those assumptions; how these changes will impact key sectors and the relative risk tolerance of key actors; how much these impacts will cost; and how to prioritize adaptations while taking into account associated risks. While descriptions of future climate certainly play an important part in answering these questions, a host of different kinds of information are also needed. Providing decisions makers with this information will frequently require climate scientists and/or service providers to collaborate with sectoral experts. The extent to which climate services are able to provide information is an important attribute of their effectiveness.

### Governance, Process, and Structure of the Climate Service

The range of actors involved, and the range of issues that must be addressed, in the development and delivery of climate services requires the development of structures that can facilitate interactions between dispersed institutional and administrative mechanisms, projects, and financial resources; it may suggest a role for private-sector services to fill the gap.^151^ In this context, the structure and governance of a climate service are important determinants of the effectiveness of the service itself. For instance, a service built on sustained dialog between users and providers is generally considered more effective than one that does not include this dialog, not only because sustained dialog is essential to transmit information between users and providers but also because sustained dialog can contribute to the creation of legitimacy and trust.^152^

The perceived objectivity of the process by which the information is shared also determines the extent to which users will engage with information. This requires effective communication between parties, transparency, and flexibility.^49^ It also requires some measure of intellectual, economic, and political independence of the groups generating knowledge—which, in some cases, may require sustained public support. While the range of funding mechanisms underwriting the climate service operations described above is diverse, many rely either on public funds; others rely on project funding and have no permanent source of support. This more precarious situation is seen to limit their effectiveness over time.^150^

The extent to which climate services engage with research is also important. The quality of climate services is linked to advances made by fundamental and applied science. As a result, strong ties between climate services and research institutions that explore relevant physical and social sciences are essential. This is true both of climate service centers and of climate service activities targeted to specific locations. Financial arrangements that sustain these links are also critically important.

### Socioeconomic Value of the Climate Service

Assessing the effectiveness of a climate service should involve some assessment of its economic value. Building from similar studies with weather information,^153^ a significant body of literature has been devoted to economic valuation, particularly with respect to seasonal climate forecasts.^37^,^154^,^155^ Unfortunately, while the notion that climate information is economically valuable has been established, questions of when this information is more or less valuable have been proven harder to resolve. Part of the difficulty associated with this is related to challenges of methodology. Determining just how to assess the value of a service is complicated, involving a range of different methodologies for assessing perceived local-level and aggregated impacts^134^; valuation information must also be put in context so that impactful climate services targeted to low-income users are not dismissed as ‘low value’. User surveys, case studies, contingent valuation methods, and empirical modeling have been used to assess the economic value of different forecast types in different decision systems and environmental and policy contexts.

In addition to the challenge of modeling a complex and unwieldy interaction with many moving parts, scholars who attempt to estimate the value of climate information are challenged by oversimplification, including attribution and a lack of attention to outcomes that are not easily measured, and a lack of explicit attention to the distribution of damages and benefits, especially the impacts of catastrophically large negative events on highly vulnerable activities or groups. There are also challenges in incorporating realistic estimates of the imperfect nature of forecast information and the extent to which they are skilful for and relevant to specific geographic regions, time horizons, and climate parameters. To accurately characterize the socioeconomic value of climate services, researchers will need to improve present indicators of skill and relevance.^131^

### Toward a More Holistic Assessment of Climate Services

The factors described above are articulated in greater detail in the relatively rich literature on the use of seasonal forecasts; they will continue to inform efforts to evaluate climate services in the future. As of yet, however, there is no agreement on the metrics or methodologies that should be used to evaluate climate services with respect to even one of the items on the list, let alone across all the design elements. Answering questions about the extent to which climate services should be supported will thus require close analysis of these various items and the trade-offs and interactions between them. Establishing effective metrics and methodologies for analysis in particular contexts, and with particular goals in mind, will be an important first step.

## GENERAL CONCLUSIONS AND AREAS FOR FUTURE RESEARCH

Climate services have gained a great deal of attention in recent years; this is reflected in the implementation of the Global Framework for Climate Services and in the rise of climate service providers in both public and private sectors and on international, national, and regional scales. Despite this growing attention, little is known about the relative effectiveness of climate services themselves or the varying institutional arrangements that support them. This paper has briefly reviewed these arrangements and put forth design elements for an evaluation framework by which we can begin to assess the relative effectiveness of climate services, including which are more successful, in what ways, and why.

In doing so, the article also highlights several areas of social science research regarding the development of climate services. This includes:

- Examining the trade-offs associated with climate service provision at different scales, as well as the role of partnerships between different organizations, and different kinds of organizations, in providing the technical and contextual expertise needed to develop effective services. Research on this topic would explore the role of the public and private sector in climate service provision, as well as the relative effectiveness of various institutional arrangements established to support climate service development and provision. The comparative advantages of international, national, and regional climate service providers in different contexts should also be explored.
- Identifying appropriate metrics and methodologies to assess climate services with respect to the various elements described in the conceptual framework. This includes developing methodologies to evaluate the extent to which climate services identify tractable problems; engage with specific decision-making contexts; tailor and communicate high-quality, useful information; establish effective governance mechanisms; and provide economic benefits to users should be documented and assessed for their relative merits. Separate methodologies should be developed for the range of climate services mentioned above; methodological guidelines should be tailored toward different types of evaluations (for instance, those geared toward learning about best practice vs assessment, and depending on the level of resources available) as well.
- Identifying, testing, and validating the metric and methodologies listed above should produce new information on the kinds of climate services that are more or less effective in specific user contexts and where to focus attention in order to improve the utility and usability of climate services. This should include questions regarding the contexts in which climate services should be supported by public versus private funds or used to foster development outcomes.
- An articulation of standards for what constitutes ‘quality’ climate information. A robust evaluation of climate services will shed light on the kinds of climate information that are more or less useful for specific decision-making contexts; this will help the climate science, and the climate service, community to develop minimum standards for what constitutes robust climate information in particular contexts. Such standards will help to distinguish between the services provided by the various types of actors named above and providing users with some measurable criteria to understand the relative quality of information they are provided. Present indicators regarding the skill of climate information should also be improved.
- It will also be necessary to develop methodologies to assess and understand trade-offs between the design elements of an evaluation framework and their relative contribution to the effectiveness of climate services in context. While it is unrealistic to expect all climate services to perform well across each of the four elements, understanding the extent to which these various items combine to create successful services will be important to decision makers. Defining a methodology to assess climate services across these range of elements will allow decision makers to recognize quick wins regarding investment in climate service development and provision, and help policymakers prioritize areas in which climate services are likely to be more effective and impactful.
- An exploration of the impact of climate services across different actors and on society as a whole. The evaluation of climate services must necessarily involve an interrogation of the extent to which the problem identified by the climate service providers is valid and important in a wider societal context. In this sense, climate services evaluations must engage with the fact that these services will necessarily privilege some groups over others. Understanding the economic and ethical implications of providing climate information to certain groups, perhaps at the expense of others, is an important part of the climate service evaluation research agenda.
